# Bilateral testicular adrenal rest tumors in a patient with nonclassical congenital adrenal hyperplasia

**DOI:** 10.1002/iju5.12299

**Published:** 2021-05-03

**Authors:** Erica C Roberts, Samantha W Nealon, Jasreman Dhillon, John B Tourtelot, Bryan McIver, Wade J Sexton

**Affiliations:** ^1^ Department of Genitourinary Oncology H. Lee Moffitt Cancer Center Tampa Florida USA; ^2^ Department of Anatomic Pathology H. Lee Moffitt Cancer Center Tampa Florida USA; ^3^ Department of Endocrinology H. Lee Moffitt Cancer Center Tampa Florida USA

**Keywords:** adrenal insufficiency, ectopic adrenal rest tumors, elevated ACTH, nonclassical CAH, TART

## Abstract

**Introduction:**

Solid testis tumors in post‐pubertal males usually represent germ cell malignancies; however, other uncommon or rare histologies must be considered.

**Case presentation:**

We present a case of an 18‐year‐old male undergoing attempted bilateral partial orchiectomies for suspected germ cell tumors. Tumor pathology, laboratory results, radiographic studies, and post‐surgical elevated adrenocorticotropic hormone levels supported the diagnosis of testicular adrenal rest tumors secondary to previously undiagnosed nonclassical congenital adrenal hyperplasia.

**Conclusion:**

Testicular adrenal rest tumors are rare in patients with nonclassical congenital adrenal hyperplasia and may be accompanied by adrenal insufficiency and hypogonadism, which can be treated with glucocorticoid therapy and testosterone replacement. Differential diagnosis of tumors is challenging but necessary for proper symptom‐based management.

**Abbreviations & Acronyms**
ACTH: adrenocorticotropic hormone
AFP: alpha‐fetoprotein
CAH: congenital adrenal hyperplasia (classical)
CBC: complete blood count
CT: computed tomography
FSH: follicle‐stimulating hormone
H&E: hematoxylin and eosin
LDH: lactate dehydrogenase
LH: luteinizing hormone
MRI: magnetic resonance imaging
NCAH: nonclassical congenital adrenal hyperplasia
OART: ovarian adrenal rest tumor
TART: testicular adrenal rest tumor

**Keynote message**

Reported here is a rare case of a male patient diagnosed and treated surgically for TARTs in the presence of previously undiagnosed NCAH. Elevated ACTH is the primary indicator and predictor for TARTs. Such tumors may be accompanied by adrenal insufficiency and hypogonadism, which can be treated with glucocorticoid therapy and testosterone replacement.

## Introduction

Mutations in the *CYP21A2* gene cause a deficiency in the 21‐hydroxylase enzyme.^1^ A deficiency or absence of this enzyme leads to a reduced synthesis of cortisol and aldosterone with subsequent elevated ACTH levels. These hormonal abnormalities are associated with CAH, although the differential is broad including Addison’s disease.

There are two main types of CAH, classical and nonclassical. Classical CAH is less common, occurring in one 1 of 16 000 live births worldwide.^2^ Classical CAH includes salt‐wasting and simple virilizing forms. In the salt‐wasting form, 21‐hydroxylase is almost entirely absent, and in the simple​ virilizing form, it is approximately 98–99% deficient.^1^ Classical CAH may be detected at birth screenings. In females, CAH presents as ambiguous genitalia. NCAH (or late‐onset CAH) is a milder form with only 50–80% 21‐hydroxylase deficiency.^1^ NCAH is the most common autosomal recessive endocrine disorder, but the exact prevalence is unknown.^2^ It has been estimated to occur in about 0.1% of the general worldwide population, although prevalence is higher in Hispanics and Yugoslavs (1–2%), and in those of Ashkenazi descent (3–4%).^3^

TARTs are a known sequela of CAH and are found in up to 94% of male classical CAH cases.^4^ They develop when adrenal rest cells are stimulated by elevated ACTH levels. Although TARTs are benign, they can affect testosterone production and lead to infertility due to tubular obstruction. In contrast to CAH, the incidence of TARTs in patients with NCAH is unknown. Herein, we report a rare case of a patient with bilateral testicular tumors subsequently discovered to be TARTs leading to the diagnosis of NCAH.

## Case report

**Fig. 1:**
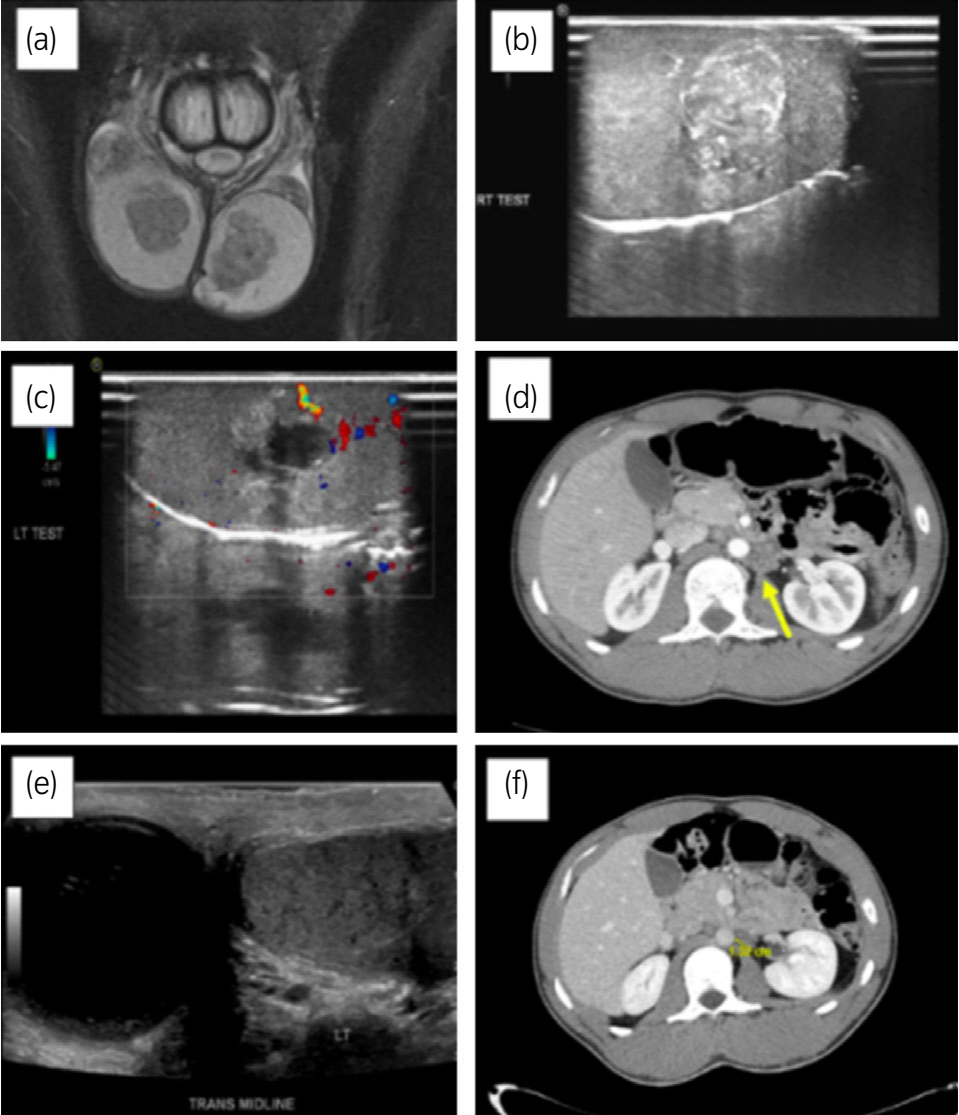
Perioperative and intraoperative imaging. Bilateral testicular masses on preoperative T1‐weighted MRI (a). Intraoperative ultrasound images of right (b) and left (c) testicles. Left para‐aortic soft tissue mass (yellow arrow) identified on CT abdomen at the time of diagnosis (d). Radiographic studies at 2 years of follow‐up demonstrate a right testicular prosthesis and a left testicle without recurrent mass on scrotal ultrasonography (e), as well as the stable appearance of a left para‐aortic soft tissue mass on CT abdomen (f).

**Fig. 2:**
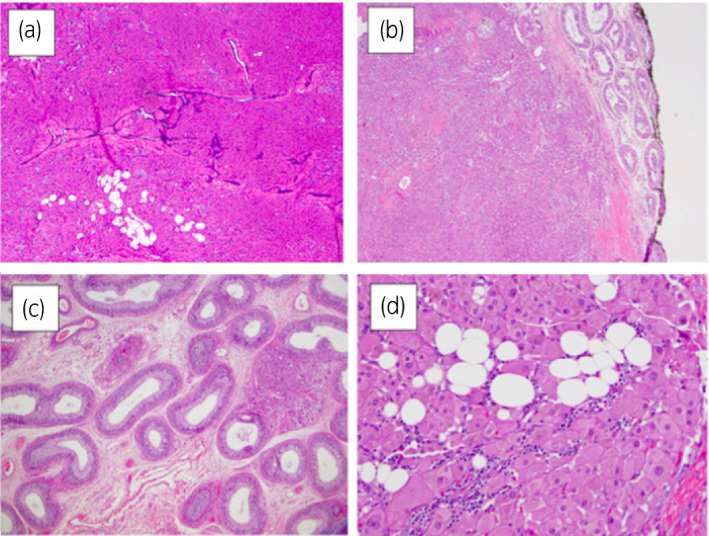
H&E‐stained, paraffin‐embedded tissue sections from TARTs. TART (4× magnification) containing adipose tissue metaplasia involving the rete testis (a) and with adjoining normal seminiferous tubules where black ink designates the surgical resection margin (b). H&E section (4×) demonstrating minute focus of a TART involving the epididymis (c). H&E section (20×) of a TART exhibiting round nuclei and abundant eosinophilic cytoplasm with lipochrome pigment, admixed with foci of metaplastic fat cells (d).

An 18‐year‐old man was referred for painful bilateral testicular tumors confirmed with scrotal ultrasonography. MRI revealed a 2.5 cm tumor and a 3.0 cm tumor in the rete testis regions of the right and left testicles, respectively (Fig. 1a). CBC, LH (4.9 mIU/mL, normal 1.5–9.3 mIU/mL), FSH (5.1 mIU/mL, normal 1.6–8.0 mIU/mL), and total testosterone (398 ng/dL, normal 249–836 ng/dL) were normal. Tumor marker beta‐HCG, AFP, and LDH were also normal. Both tumors subjectively progressed in size during the time between initial evaluation and surgery. Bilateral partial orchiectomies were attempted (Fig. 1b,c). The right was converted to a radical orchiectomy with prosthesis due to intraoperative concerns regarding testis viability. A partial orchiectomy was successfully completed on the left. Intraoperative frozen sections were not conclusive for germ cell, Leydig cell, or another histology. Pathologic evaluation of both tumors revealed the proliferation of tumor cells throughout the testicular parenchyma and rete testis, with nodules ranging from <1 to 10 mm, a low proliferation index (Ki67 positive in 1% of cells) and negative p53. No features of malignancy such as necrosis, mitotic figures, or pleomorphism were present (Fig. 2). A presumptive diagnosis of Leydig cell tumors *vs* TARTs was suggested.

CT scans of the abdomen and pelvis revealed a soft tissue density measuring 2.1 × 1.7 cm in the left para‐aortic region (Fig. 1d). Also noted were slightly enlarged inter‐aortocaval nodes and a small left adrenal nodule. An image‐guided biopsy of the para‐aortic mass was nondiagnostic.

A postoperative endocrine evaluation revealed markedly elevated ACTH levels (4322 pg/mL) yet normal cortisol levels. The patient’s clinical and radiographic findings, testis pathology, and elevated ACTH level, all supported a diagnosis of NCAH and the presence of TARTs. The postoperative endocrine evaluation also revealed an LH level of 21.5 mIU/mL, FSH of 22.9 mIU/mL, and a low testosterone level (81 ng/dL). Borderline adrenal insufficiency and moderate hypogonadism were effectively managed with hydrocortisone and testosterone supplementation. Larger than average doses of hydrocortisone were necessary to maintain the ACTH at a desired level (under 100 pg/mL).

Follow‐up scrotal U/S and CT scans 2 years post‐surgery revealed a right testicular prosthesis, a partial left testicle containing no new masses, and a stable para‐aortic soft tissue mass (Fig. 1e,f).

## Discussion

TARTs are far less prevalent in patients with NCAH compared to CAH. Although our patient was not tested for 21‐hydroxylase deficiency for confirmation, clinical findings supported this diagnosis. In addition, we suspected adrenal rest tissue in the left para‐aortic region, although an image‐guided biopsy was inconclusive. Females with CAH and NCAH have rarely experienced OARTs.^4^ Other studies of NCAH have revealed ectopic adrenal rests not only in testes (or ovaries) but also in the spermatic cord, celiac plexus, broad ligaments, liver, kidneys, spinal cord, and corti of adrenal glands.^4^, ^5^, ^6^ Reports on patients with NCAH with ectopic adrenal rest tumors are detailed in Table 1.

**Table 1:** Reported cases of patients with NCAH with ectopic adrenal rest tumors

	Our patient	Ref. [4] Case 1	Ref. [4] Case 2	Ref. [5]	Ref. [6]
Sex at birth	Male	Female	Male	Male	Female
Age at diagnosis	18	41	29	41	55
TART/OART	Yes	No	Yes, bilateral	No	No
Tumors outside testicular/ovarian region	Left infrahilar para‐aortic region	Bilateral adrenal cortex	No	Left adrenal cortex, left adrenal hilum	Liver
Adrenal hyperplasia	No	Yes	Yes, bilateral	Yes	No

The cells with potential to become TARTs originate during early gestation, when cells with the intended role of producing steroids in the adrenal cortex and gonads differentiate from the surrounding coelomic epithelium. Some cells that migrate to the gonads retain ACTH responsiveness, having the potential to become TARTs.^7^ When there is a sustained elevation of ACTH in patients with CAH and NCAH, adrenal rest cells are overstimulated and develop into tumors.

Differentiating TARTs from Leydig cell tumors is necessary as prognosis and treatment are vastly different. Whereas TARTs are benign, Leydig cell tumors can have more serious implications, as 10% are malignant and uniformly fatal in the setting of metastatic progression.^4^, ^8^ As a starting point for differentiation, TARTs are usually bilateral (>80%), whereas Leydig cell tumors are usually unilateral (bilateral in only 3%).^4^ TARTs are composed of large eosinophilic cells with abundant cytoplasm. Other TART‐identifying features include: fibrous septae, lipochrome pigment, adipose metaplasia, nuclear atypia, low mitotic activity, and absence of Reinke crystals. While formulating a differential diagnosis for a patient with bilateral testis tumors, it is important to keep in mind that coexistence of tumor types is possible as is synchronous germ cell malignancies.^9^ In addition, although the patient’s discomfort and tumor size progressed over a short period of time prior to surgery, it is possible that we could have staged right and left operations given the difficulty with diagnosis on intraoperative frozen section and the inability to complete a partial orchiectomy for the right‐sided mass.

Table 2 shows the characteristics of CAH compared with NCAH. Aside from testicular tumors and mild acne, our patient lacked many symptoms typical of NCAH, as he had normal puberty at age 10–11, normal stature, and normal secondary sexual characteristics. Since both CAH and NCAH are inherited recessively, family history is important in identifying patients for routine ACTH screening or testing for *CYP21A2* mutations. Our patient’s family history was negative. However, when early diagnosis is possible, monitoring ACTH levels and optimizing glucocorticoid therapy may be the only way to effectively prevent TART development or size progression to minimize any negative effects on the reproductive system.^10^

**Table 2:** Classical *vs* NCHA

	CAH	NCAH
Onset		
Initial diagnosis	Infancy – newborn screening	Usually during late adolescence or early adulthood
	Based on blood work, biochemical, and genetic testing confirmation	Often prompted by tumor findings on physical exam
Extent of 21‐hydroxylase deficiency	Simple virilizing: 98–99% deficiency	50–80% deficiency
	Salt‐wasting: near complete deficiency	
Symptoms†	Precocious puberty	Precocious puberty
	*TARTs (males) in up to 94% of cases*	*Early, rapid growth and eventual short stature*
		*Early beard growth (males) or hirsutism (females)*
		*TARTs (males) infrequent but possible; OARTs (females); Potential adrenal rest tumors in other areas of body*
	Infertility	Infertility
	Acne	Acne
	*Ambiguous genitalia (females)*	
	Elevated ACTH	Elevated ACTH
	Excessive androgen production	Excessive androgen production
	Low cortisol levels	Low cortisol levels
	Low aldosterone levels	Low aldosterone levels
	Hypoglycemia	Hypoglycemia
	Irregular periods (females)	Irregular periods (females)
		*Male‐pattern baldness (females)*
	Enlarged penis and small testes (males)	Enlarged penis and small testes (males)
	Adrenal hyperplasia	Adrenal hyperplasia
	*Low sperm count (males)*	*Normal sperm count (males); Low sperm count more likely if TARTs are present*
	Advanced bone age	Advanced bone age
Treatment†	Removal of tumors (if present)	Removal of tumors (if present)
	Steroids	Steroids
	Supplemental hormones	Supplemental hormones
	Surgical correction of ambiguous genitalia (females)	
Life expectancy	Normal life expectancy with proper treatment	Normal life expectancy with proper treatment
	Psychological support may be needed	Psychological support may be needed

† Some patients with mild NCAH may never experience symptoms or require treatment. Carriers do not show any symptoms or require treatment. More subtle symptom differences can exist between the types of classical CAH. (Table adapted from Refs [1, 11, 12, 13].)

## Conclusions

Adrenal rest tumors are rare in patients with NCAH but can occur throughout the pelvic and abdominal cavities. TARTs typically do not present until late adolescence or adulthood and can be the first sign of NCAH. Differential diagnosis is challenging but necessary to direct management and surveillance. Elevated ACTH is a predictor of TARTs. Family history is also relevant. Prediction or early detection of TARTs could allow for treatment prior to infertility. TARTs can respond to glucocorticoid therapy used to manage adrenal insufficiency, and testosterone replacement has been successful for patients with hypogonadism.

## Conflict of interest

The authors declare no conflict of interest.
